# Periocular Involvement in Trigeminal Trophic Syndrome

**DOI:** 10.7759/cureus.68738

**Published:** 2024-09-05

**Authors:** Sonia Peñarrocha-Oltra, Alfonso V Perez, Kaveh Vahdani

**Affiliations:** 1 Ophthalmology, Moorfields Eye Hospital NHS Foundation Trust, London, GBR

**Keywords:** cerebrovascular accident, herpes zoster, neuralgia, neuropathy, trigeminal trophic syndrome

## Abstract

Trigeminal trophic syndrome (TTS) is an uncommon condition resulting from trigeminal nerve damage, characterized by persistent facial ulceration, loss of sensation, and paresthesia within the trigeminal dermatome, with ala nasi involvement being a key feature. Lesions develop from repeated self-inflicted manipulation and trauma of the dysesthetic skin. This report details three cases of TTS, highlighting periocular changes, with etiologies varying from cerebrovascular accidents to herpes zoster ophthalmicus.

## Introduction

Trigeminal trophic syndrome (TTS) is an uncommon condition that occurs after a peripheral or central injury to the trigeminal system [1,2]. It is characterized by a triad of anesthesia, paresthesia, and secondary persistent or recurrent unilateral facial ulceration, typically crescent-shaped and involving the ala nasi [1,3]. The most common causes are ischemic damage involving the posterior encephalic vascular territory and ablative treatments for trigeminal neuralgia [3], with other potential causes including acoustic neuroma, herpes zoster, postencephalitis, posterior fossa tumors, trauma, and syringobulbia [4].

Diagnosis is clinical, made by excluding other necrotizing or ulcerative conditions, with histology being non-specific [5]. Management is challenging, requiring a multidisciplinary approach, emphasizing patient education, and counseling to reduce skin manipulation [5]. TTS and its associated periocular changes are scarcely featured in the ophthalmic literature, remaining relatively underrecognized. The authors review the literature and describe three cases of TTS with ophthalmic involvement caused by stroke (two cases) and herpes zoster ophthalmicus (one case). This report adheres to the principles of the Declaration of Helsinki.

## Case presentation

Case 1

**Figure 1:**
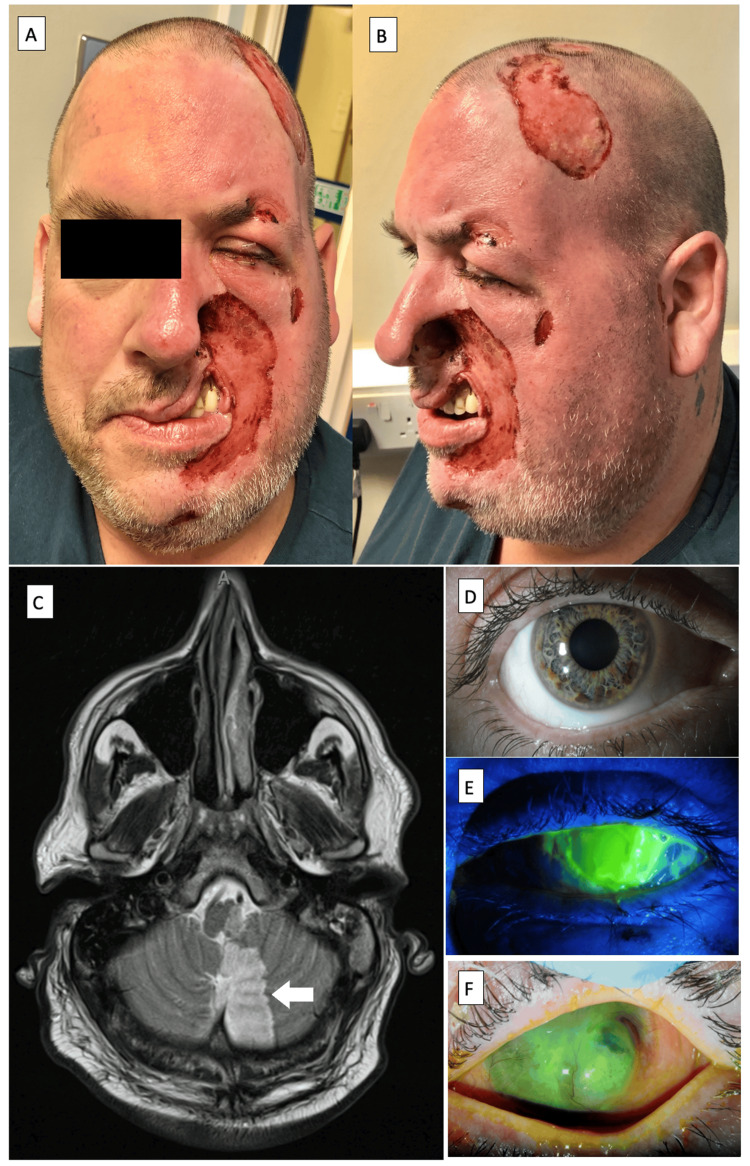
Images of case 1 (A,B) Clinical appearance at presentation with unilateral ulcers in the trigeminal territory, crescent-shaped, with ala nasi involvement, and sparing of the tip of the nose. (C) MRI T2/FLAIR sequence showed a large left posteroinferior cerebellar artery (PICA) territory infarct, affecting the inferomedial left cerebellar hemisphere and left side of the medulla. (D) Right eye showed unremarkable findings. (E) Left pancorneal epithelial defect, stromal thinning, and anesthesia. (F) Two months after treatment with topical antibiotics, an amniotic membrane graft, and a temporary tarsorrhaphy, the epithelial defect had completely healed, but the cornea remained opaque with superotemporal descematocele.

A 51-year-old male with a history of a left posteroinferior cerebellar artery (PICA) infarct two years ago presented with a total left corneal epithelial defect, stromal thinning, and anesthesia associated with ipsilateral facial dysesthesia and crescent-shaped ulcers in the trigeminal territory (Figure 1). Despite awareness of his habitual scratching, he felt compelled by the dysesthesia. After treatment with an amniotic membrane graft, temporary tarsorrhaphy, and topical antibiotics, steroids, and lubricants, the corneal epithelial defect healed, and he was referred to the maxillofacial department for reconstructive surgery consideration and to psychiatry for counseling.

Case 2

**Figure 2:**
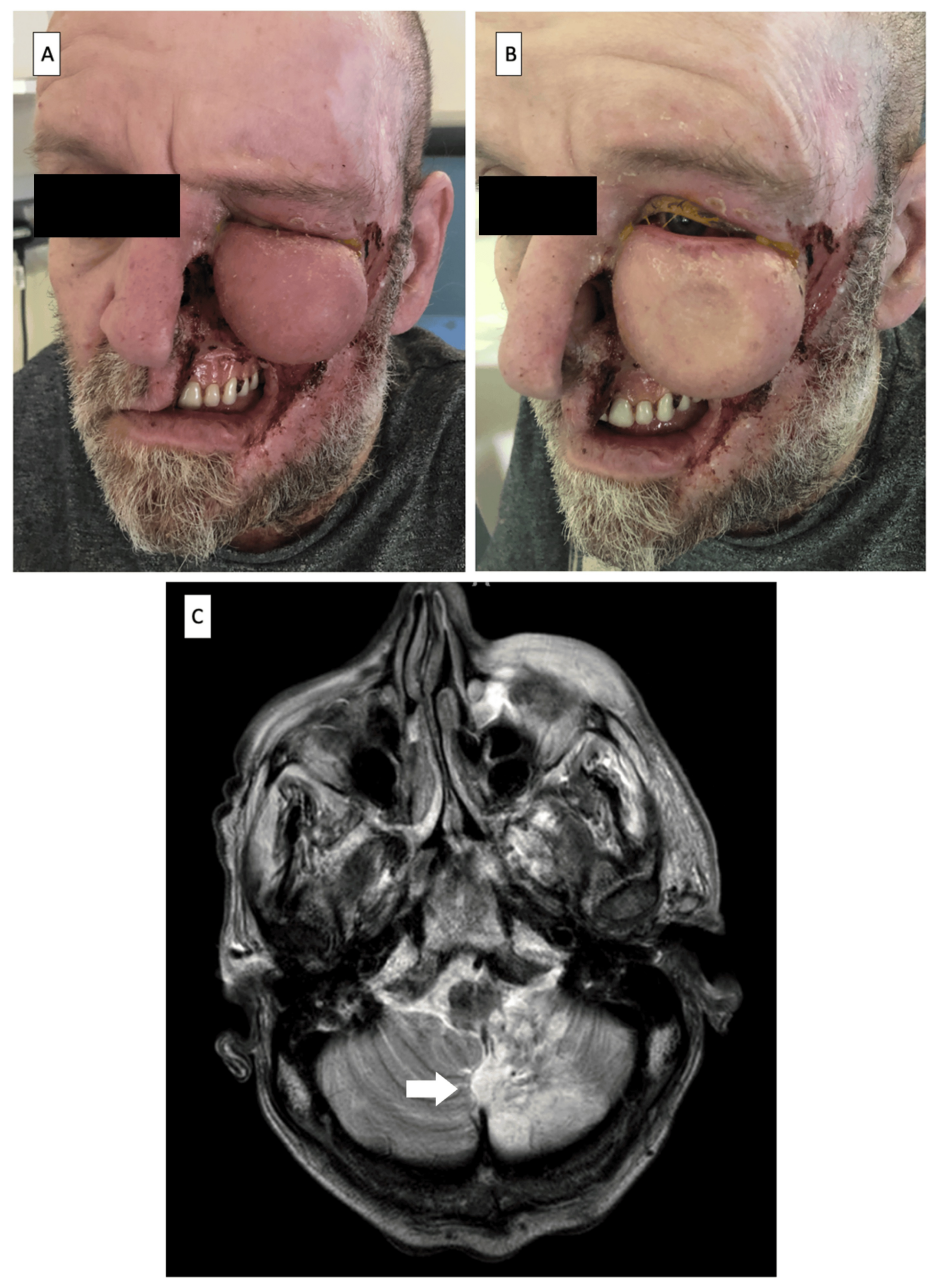
Images of case 2 (A,B) Mid-facial ulcerations in the left maxillary nerve dermatome, extensive soft tissue loss, absent left ala nasi but sparing of the tip of the nose, and severe left lower lid pitting lymphoedema. (C) MRI T2/FLAIR sequence showed established left PICA territory infarct and left malar soft tissue edema.

A 56-year-old male with a history of type 2 diabetes and a left cerebellar and brainstem infarction one year prior was referred with a six-week history of a lump in his left lower lid. He had recurrent mid-facial ulcerations in the maxillary nerve dermatome, extensive soft tissue loss, absent left ala nasi, severe left lower lid lymphoedema (Figure 2), and admitted lesion picking. Biopsies revealed non-specific chronic inflammation without evidence of infection, vasculitis, or neoplasm. Although planned for reconstructive surgery by plastic surgeons, he succumbed to systemic comorbidities two years later.

Case 3

A 78-year-old diabetic and hypertensive male presented with left herpes zoster ophthalmicus, treated with oral acyclovir and topical antibiotics. One month later, he developed post-herpetic neuralgia and facial ulcerations, managed with morphine, pregabalin, duloxetine, and capsicum cream. He had a large cutaneous ulcer on his left upper lid and scalp, along with lagophthalmos and left upper lid entropion, due to persistent and recurrent facial ulcerations. Refusing surgery, he opted for eyelash epilation, lubricants, and topical antibiotics.

## Discussion

TTS, resulting from trigeminal nerve damage, is a rare condition with significant morbidity, primarily diagnosed clinically, and should be suspected in patients with persistent or recurrent facial ulceration, along with anesthesia and paresthesia in the trigeminal distribution [6]. Lesions typically exhibit a crescent shape, often involving the ala nasi due to innervation disparities between trigeminal branches V1 and V2, with the nasal tip usually spared due to sensory innervation by the external branch of the anterior ethmoid nerve [3]. 

The underlying pathologic mechanism of TTS remains unclear, as trigeminal nerve damage does not always result in the syndrome. The mean age of presentation is reported at 57, with a female predominance ratio of 2.2:1 [7], contrasting with all our cases being males. The latency period between the trigeminal nerve insult and the onset of ulceration varies from weeks to decades [7]. Our two patients with stroke had a one- to two-year latency, while the third case with herpes zoster ophthalmicus had a four-week latency.

Patients with TTS often have psychiatric comorbidities, such as reiterated self-destructive behavior, mood dysfunction, anxiety, Alzheimer’s disease, or obsessive-compulsive disorder (OCD) [4,5]. In the cases presented, the most frequent comorbidity was diabetes mellitus type 2.

Histological findings in TTS ulcers are nonspecific, but biopsy aids in excluding malignancy, infection, or vasculitis [5]. Differential diagnoses also include dermatitis artefacta, distinguished by the absence of a neurological deficit [3]. Therefore, confirming nerve injury and evaluating pain, thermal perception, and corneal sensitivity are essential for diagnosis.

While there is no standard therapeutic protocol for TTS, management is based on four main strategies: early diagnosis, behavioral modifications, wound care, and pharmacological treatment [5,6]. Patient counseling and education are crucial in understanding and preventing the harmful effects of physical manipulation of the skin. Occlusive dressings can reduce recurrent trauma, and topical lubricants, antibiotics, and autologous serum may be required in cases with corneal involvement or exposure [4]. Surgical interventions for ocular protection, such as temporary tarsorrhaphy as in our first case, may be considered; however, major surgeries might not always yield success, especially if the underlying neurological pathology and skin manipulation have not been adequately addressed. Our patients received psychological support and multidisciplinary care involving dermatologists, maxillofacial, and plastic surgeons. Unfortunately, the second and third patients passed away two and eight years post-diagnosis of TTS, respectively.

## Conclusions

Our case series aims to raise awareness of TTS and its ophthalmic manifestations among ophthalmologists, highlighting its rarity and the lack of a specific cure. Correct diagnosis is crucial, emphasizing the need to rule out neoplastic and infectious causes before confirming TTS. Clinical examination showing facial ulcerations in the trigeminal dermatome as well as ala nasi involvement and sparing of the tip of the nose help to establish the diagnosis of TTS.
